# The development of pevonedistat in myelodysplastic syndrome (MDS) and acute myeloid leukemia (AML): hope or hype?

**DOI:** 10.1177/20406207221112899

**Published:** 2022-07-22

**Authors:** Anson Snow, Joshua F. Zeidner

**Affiliations:** Lineberger Comprehensive Cancer Center, University of North Carolina School of Medicine; Division of Hematology, Department of Medicine, University of North Carolina School of Medicine, Chapel Hill, NC, USA; Lineberger Comprehensive Cancer Center, University of North Carolina School of Medicine; Division of Hematology, Department of Medicine, University of North Carolina School of Medicine, 170 Manning Drive, POB, 3rd Floor, CB #7305, Chapel Hill, NC 27599, USA

**Keywords:** Acute myeloid leukemia (AML), myelodysplastic syndrome (MDS), NEDD8 activating enzyme (NAE) inhibitor, pevonedistat

## Abstract

Myelodysplastic syndrome (MDS) is a clonal hematopoietic stem cell disorder clinically defined by cytopenias, bone marrow failure, and an increased risk of progressing to acute myeloid leukemia (AML). Traditionally, first-line treatment for patients with higher-risk MDS has been hypomethylating agents (HMAs). However, these agents have modest clinical activity as single agents. A one-size-fits-all treatment paradigm is insufficient for such a heterogeneous disease in the modern era of precision medicine. Several new agents have been developed for MDS with the hopes of improving clinical outcomes and survival. Pevonedistat is a first-in-class, novel inhibitor of neuronal precursor cell-expressed developmentally down-regulated protein-8 (NEDD8) activating enzyme (NAE) blocking the neddylation pathway leading to downstream effects on the ubiquitin–proteosome pathway. Pevonedistat ultimately leads to apoptosis and inhibition of the cell cycle in cancer cells. Studies have demonstrated the safety profile of pevonedistat, leading to the development of multiple trials investigating combination strategies with pevonedistat in MDS and AML. In this review, we summarize the preclinical and clinical rationale for pevonedistat in MDS and AML, review the clinical data of this agent alone and in combination with HMAs to date, and highlight potential future directions for this agent in myeloid malignancies.

## Introduction

Myelodysplastic syndrome (MDS) is a myeloid malignancy that generally affects the elderly, with a median age at diagnosis of 76 years in the United States.^1^ In fact, more than 85% of patients diagnosed with MDS are 60 years and above.^2^ The estimated incidence of MDS is 4 per 100,000^3^ in the United States, though this likely underrepresents the true prevalence of this disease.^4^ MDS is a clinically heterogeneous disease noted by ineffective hematopoiesis leading to cytopenias that can cause significant transfusion and supportive care needs.^5^ In addition, MDS lies on a disease continuum with acute myeloid leukemia (AML), the latter defined as ⩾20% myeloblasts in the peripheral blood (PB) or bone marrow (BM).^6^ Over time, MDS can develop an evolution of clonal abnormalities leading to AML.^7^ Thus, the goal of treatment for MDS patients is to improve quality of life through symptom control and prevent disease progression and mortality.^8–10^

## Diagnosis of MDS

Traditionally, MDS is suspected in a patient with cytopenias where other etiologies have been ruled out, prompting a BM biopsy for further evaluation. MDS diagnosis based on the World Health Organization (WHO) definition of MDS includes the following criteria: number of dysplastic lineages, cytopenias, ring sideroblasts, BM or PB blast, and cytogenetics. Using these characteristics, a patient’s MDS can be placed into one of six main subtypes: MDS with single lineage dysplasia (MDS-SLD), MDS with multilineage dysplasia (MDS-MLD), MDS with isolated del(5q), MDS with ring sideroblasts (MDS-RS), MDS with excess blasts (MDS-EB), and MDS, unclassified (MDS-U).^6^ Once a diagnosis of MDS has been established, patients can be risk-stratified using several scoring systems. The International Prognostic Scoring System (IPSS), published in 1997 and revised in 2012 (IPSS-R), is the most widely used risk criteria for newly diagnosed MDS.^11,12^ The IPSS-R uses blood counts, BM blast percentage, and cytogenetics to stratify MDS patients into five risk categories: very low, low, intermediate, high, and very high risk. Molecular genetic testing can supplement IPSS-R scores as specific mutations can affect the clinical course of MDS. Generally, patients are subdivided into lower risk (very low, low, and some intermediate for IPSS-R) and higher risk (some intermediate, high, and very high IPSS-R) to determine treatment paradigms.^13^ However, lower-risk and higher-risk MDS treatment arms are an oversimplification as a complete clinical picture including the severity of cytopenias, transfusion needs, age, comorbidities, mutation profile, prior treatment, allogeneic stem cell transplantation (alloSCT) eligibility, patient’s goals, in addition to risk stratification scores are also used to determine optimal treatment options.^14^

## Clinical outcomes of MDS and unmet needs

Median overall survival (OS) for very low and low-risk MDS can range from 8.8 to 5.3 years without therapy, and some patients may have relatively indolent disease for months to years without the need for therapeutic intervention.^15^ Thus, treatment options for lower-risk MDS are predicated on improving symptom burden and quality of life and delaying or preventing progressive disease. Treatment options for lower-risk MDS include erythroid stimulating agents (ESAs), thrombopoietin agonist (TPO), luspatercept, lenalidomide, immunosuppressive therapy, and hypomethylating agents (HMAs). The treatment paradigm for lower-risk MDS is individualized based on a patient’s clinical characteristics and genomic profile/the World Health Organization (WHO) classification.^16^ In contrast, median OS for high and very high-risk MDS ranges from 1.6 to 0.8 years without treatment.^15^ Due to a poorer prognosis associated with higher-risk MDS, standard frontline treatment for higher-risk MDS involves the administration of HMA’s [azacitidine (AZA), decitabine (DEC), or oral decitabine-cedazuridine (C-DEC)]. AZA given intravenous (IV) or subcutaneously (SQ) and DEC given IV were approved for the treatment of MDS by the US Food and Drug Administration (FDA) in 2004 and 2006, respectively.^17^ Both agents are structural analogs of pyrimidine nucleoside cytosine in which the carbon in the aromatic ring at the 5’ position is substituted with nitrogen. After cellular uptake, AZA and DEC are incorporated into the DNA leading to inhibition of DNA methylation and reactivation of silenced tumor suppressor genes that cause tumor cell apoptosis/senescence.^18^ Comparing these two HMAs, randomized trial data are lacking in demonstrating the superiority of either AZA *versus* DEC.^19^ There is some expert consensus that AZA may be preferable to DEC given the phase III AZA-001 study found AZA significantly improved OS compared to conventional care regimens (best supportive care, low-dose cytarabine, or intensive chemotherapy) of 24.5 months *versus* 15 months, respectively,^20^ whereas, low-dose DEC compared with best supportive care had no significant difference in OS with 10 months *versus* 8 months, respectively.^21^ However, it must be noted that the dose of DEC used in this study was 15 mg/m^2^ IV three times a day for 3 days in 6-week cycles rather than the current FDA-approved dosing schedule of DEC 20 mg/m^2^/day IV for 5 days every 4 weeks.^22^ In 2020, the FDA approved oral C-DEC for the treatment of MDS.^17^ C-DEC is a combination of DEC and cedazuridine, a synthetic cytidine deaminase (CDA) inhibitor, which decreases the metabolism of oral HMAs allowing for therapeutic levels.^23^ Although the survival benefit of C-DEC is still immature in MDS, the randomized cross-over phase III ASCERTAIN study reached its primary endpoint demonstrating that C-DEC delivers similar pharmacologic levels of DEC compared to IV DEC, thus providing another HMA treatment option in MDS.^24^ Despite these HMA options, only 50% of patients will benefit from treatment, with many responders relapsing within 2 years.^18^ Of those who fail HMA, outcomes are poor, with a median OS of 5.6 months.^25^

In addition, eligible higher-risk MDS patients are evaluated for alloSCT as this is the only curative treatment of MDS.^9,26^ Unfortunately, approximately 10% of higher-risk MDS patients are able to proceed to alloSCT despite the improved outcomes associated with alloSCT in higher-risk MDS.^27–29^ In the last decade, several seminal studies have investigated the role of molecular markers in MDS and noted mutated genes correlate with clinical outcomes.^30,31^ Some of the common mutations seen in MDS include: splicing factors (*SF3B1*, *SRSF2*, *U2AF1*, and *ZRSR2*), DNA methylation (*TET2*, *DNMT3A*, and *IDH1/2*), histone modification (*ASXL1*, *EZH2*, *BCOR*, and *EP300*), cohesion components (*STAG2*, *RAD21*, *SMC1A*, and *SMC3*), transcription factors (*RUNX1*, *ETV6*, *CUX1*, and *GATA2*), signal transduction (*CBL*, *JAK2*, *NRAS*, *KRAS*, *MPL*, *NF1*, *PTPN11*, *KIT*, and *FLT3*), and p53 (*TP53* and *PPM1D*).^32^ As more data are collected, mutation profiles are increasingly being incorporated in clinical prognosis. Given MDS disease heterogeneity and its distinct biologic features, the idea of a one-size-fits-all treatment paradigm is clearly suboptimal. The development of novel investigational strategies provides optimism for an individualized treatment approach for MDS in the next decade and beyond.

## Pevonedistat: mechanism of action and biology

Normal cellular function and homeostasis depend on the ubiquitin-proteosome system (UPS) and its ability to tag proteins for degradation through a process called ubiquitination. Briefly, there are several steps in the ubiquitination pathway. First, ubiquitin is activated through an ATP-dependent manner by the ubiquitin-activating enzyme (E1). The ubiquitin-conjugating enzyme (E2) then binds to the activated ubiquitin-E1 complex initiating the transfer of ubiquitin from E1 to E2. Ubiquitin is then transferred from E2 to the ubiquitination ligase (E3), which conjugates ubiquitin to a target protein substrate.^33^ Finally, the 26 S proteasome identifies and degrades proteins that the ubiquitin pathway has tagged.^34^ In cancer cells, the UPS is deregulated and usually has increased proteasome activity compared to normal cells.^35^ These findings led to the development of one of the first proteasome inhibitors, bortezomib (Velcade).^36^ Although proteasome inhibitors have demonstrated clinical activity in lymphoma and myeloma-related disorders, this did not translate to other malignant hematologic diseases such as AML, MDS, and acute lymphoblastic leukemia (ALL).^37^

Owing to the success of bortezomib, further investigations into the UPS led to a deeper understanding of the pathway and related enzymes that were potential therapeutic targets such as E3. E3 includes hundreds of ligases that can be subdivided into three classes based on their structural domains and mechanism of ubiquitin transfer to a substrate protein: HECT (homologous to the E6-AP carboxy terminus), RING (Really Interesting New Gene), and the RING-between-RING (RBR).^38,39^ Ring E3s are the most abundant of the ubiquitin ligases and includes a subclass called Cullin-RING ligases (CRLs).^40^ These CRLs are multi-subunit ubiquitin ligases involved in the ubiquitination and degradation of around 20% of all eukaryotic cellular proteins that control cell cycle progression, DNA repair, and signal transduction.^41^ Notably, CRL activity is regulated by neddylation and deneddylation, as described below.

Neddylation is mechanistically similar to ubiquitination but is a post-translational modification process that uses neuronal precursor cell-expressed developmentally down-regulated protein-8 (NEDD8), a ubiquitin-like molecule, rather than ubiquitin to modify proteins. The first step of the neddylation process is activation of NEDD8 by NEDD8-activating enzyme (NAE) and NEDD8 E2 (UBE2M/F) with subsequent conjugation to substrate proteins by NEDD8 E3 ligases.^42,43^ Neddylated substrates include the cullin family proteins, which assemble to make CRLs and non-cullin proteins that may regulate various functions including tumor suppression, oncoproteins, receptor proteins, and transcriptional regulators.^44^ Notably, neddylation pathways are involved in tumorigenesis as they are upregulated in several malignancies, helping to promote cell growth and evasion of programmed cell death through the degradation of tumor suppressor proteins regulated by CRLs.^41,45,46^ Based on these findings, the neddylation pathway became an active area of interest for cancer drug development and led to the development of a novel inhibitor against NAE, pevonedistat (MLN4924 or TAK-924).

Pevonedistat is a first-in-class, novel inhibitor of NAE and has been investigated alone and in combination with AZA in MDS and AML.

**Figure 1.**
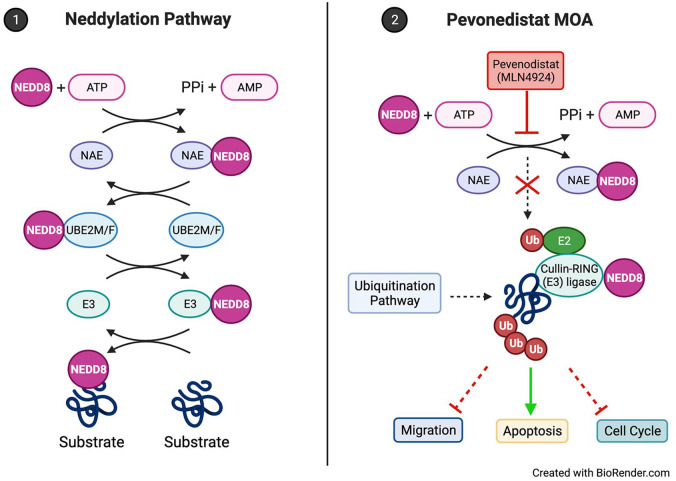
(1) The canonical neddylation enzymatic cascade where NEDD8 is conjugated to substrates. ATP-dependent NAE activates NEDD8 and is loaded onto UBE2M/F. NEDD8 is then conjugated to a substrate by E3. (2) Pevonedistat is a small-molecule inhibitor that acts as an AMP mimetic to block the NAE adenylation active site, terminating the neddylation enzymatic cascade. Downstream effects lead to the inhibition of cancer cell migration, disruption of the cell cycle, and activation of apoptosis pathways. AMP, adenosine monophosphate; ATP, adenosine triphosphate; E2, ubiquitin-conjugating enzyme; NAE, NEDD8-activating enzyme; PPi, pyrophosphate; Ub, ubiquitin; UBE2M/F, ubiquitin-conjugating enzyme E2 M/*F* (NEDD8-conjugating enzyme).

Pevonedistat acts as an adenosine monophosphate (AMP) mimetic that binds to the NAE adenylation active site leading to the termination of the neddylation pathway. This indirectly inhibits CRL activity causing cell cycle arrest, inhibiting migration, and inducing apoptosis in cancer cells (Figure 1).^42,43,47^ Several preclinical studies have shown the efficacy of pevonedistat in both solid^48–50^ and hematologic malignancies.^51–53^ Specifically, inhibition of NAE was found to induce cell death and/or cell cycle arrest in AML cells leading to decreased transcription of NFκB genes leading to an increase of reactive oxygen species (ROS), DNA damage, and eventual apoptosis.^51^ Furthermore, pevonedistat was shown to decrease AML blast-cells and stem cell populations with minimal effect on normal hematopoietic cells.^54^ Thus, the preclinical anti-leukemic efficacy of pevonedistat paved the way for future clinical trials.

## Pevonedistat single-agent safety and efficacy

Owing to the promising preclinical data of pevonedistat in AML, an open-label, phase 1 dose-escalation study (NCT00911066) of pevonedistat was investigated in adult patients with AML or high-grade MDS.^55^ The primary objective of this study was assessing a maximally tolerated dose (MTD) with pharmacokinetics (PK) and pharmacodynamics as secondary objectives. The study included 53 patients, 50 of whom had AML and 3 high-grade MDS. Pevonedistat was given as a 60-minute IV infusion on days 1, 3, and 5 (schedule A) or days 1, 4, 8, and 11 (schedule B), with an initial starting dose at 25 mg/m^2^ up to 147 mg/m^2^. Each cycle was given every 21 days.^56^ Prior phase 1 data for pevonedistat in solid tumors demonstrated severe liver injury with continuous dosing as a dose-limiting toxicity (DLT). Thus, intermittent dosing was explored to mitigate hepatic toxicity.^57^ The schedule B dosing was based on laboratory data showing a direct relationship between pevonedistat exposure and response with the hypothesis that higher cumulative doses would improve responses.^51^ In addition, phase 1 data of pevonedistat treatment for lymphoma and myeloma did not show drug accumulation between doses for a 1-, 4-, 8-, and 11-day regimen, and only one DLT was reported at the starting dose escalation of 110 mg/m^2^ and a reported MTD of 196 mg/m^2^.^58^

Toxicities for the schedule A regimen were reported at the 78 mg/m^2^ with reversible grade 3 elevation in transaminases noted in cycle 1 for one patient and sepsis, elevated transaminases, and multiorgan failure in cycle 2 for another. Thus, the MTD for schedule A was found to be 59 mg/m^2^. For schedule B, two patients developed DLT leading to multiorgan failure with a 147 mg/m^2^ dose leading to the first dose de-escalation to 110 mg/m^2^. Two additional events, a multiorgan failure DLT and deadly fungal pneumonia unrelated to pevonedistat, led to a second dose reduction to 83 mg/m^2^ with no further DLTs reported at this dose. Thus, the MTD for schedule B was determined to be 83 mg/m^2^.^56^

The most common adverse effects (AEs) from the 53 patients combined from schedule A and B were pyrexia (53%), diarrhea (43%), febrile neutropenia (36%), chills (36%), decreased appetite (34%), fatigue (34%), edema (32%), nausea (32%), dyspnea (30%), dizziness (28%), myalgia (28%), vomiting (25%), cough (25%), elevated aspartate transaminase (23%), elevated alanine transaminase (23%), headache (23%), epistaxis (23%), and rales (21%), though the majority were grades 1 and 2. The most common ⩾3-grade AEs from the 53 patients combined from schedule A and B were thrombocytopenia (8%), febrile neutropenia (4%), elevated aspartate transaminase (4%), hypoxia (4%), hypotension (4%), multiorgan failure (4%), and fatigue (4%). One death from sepsis at 78 mg/m^2^ for schedule A and two deaths from multiorgan failure for schedule *B* (one for 110 mg/m^2^ and 147 mg/m^2^, respectively) were reported.^56^

The PK of pevonedistat demonstrated a time of maximum concentration (T_max_) to be around 1 hour with a maximum concentration (C_max_) to be proportionally related to the dose given at infusion. Furthermore, pevonedistat had a bi-exponential decrease in plasma levels with detectable concentrations noted up to 24 hours post-infusion for doses of 25 and 33 mg/m^2^ and up to 48 hours post-infusion doses at 44 mg/m^2^ and up, with an estimated half-life of 12 hours. In addition, eight mRNA transcripts expression levels (*ATF3*, *GCLM*, *GSR*, *AGPAT9*, *NQ01*, *SLC7A11*, *SRXN1*, and *TXNRD1*) were measured to assess the pharmacodynamics of pevonedistat. Notably, it was discovered that all doses could induce transcription levels of the eight mRNA of interest.

The overall response rates (ORRs) for schedule A pevonedistat at or below MTD was 17% (4/23) with two complete remissions (CRs) and two partial remissions (PRs), while the ORR for schedule B was 10% (2/19) with two PRs.^56^

## Preclinical rationale of pevonedistat + AZA

Based on the preliminary data from the phase 1 single-agent pevonedistat trial, further preclinical studies were performed to find potential therapeutic combinations with pevonedistat. Using a high-throughput viability screen against AML cells, 40 different agents were investigated. DEC and AZA, HMAs, were synergistic with pevonedistat. The combination of an HMA and pevonedistat increased DNA damage and cell death compared to single agents alone in *in vitro* studies.^59^ Also, combination therapy of AZA and pevonedistat led to tumor regression in two AML mouse xenografts with AZA-resistant cell lines, whereas AZA alone had minimal effects providing further evidence for synergy between AZA and pevonedistat.^59^

Proteomic profiling of pevonedistat targets in AML cell lines show that ribonucleotide reductase (RR), which is involved in DNA synthesis and repair, is elevated after treatment. RR comprises two dimeric subunits, RRM1 and RRM2, and overexpression is believed to be a mechanism for resistance to cytotoxic nucleoside analogs and specifically AML resistance to cytarabine.^60^ In addition to being an HMA, AZA has been shown to downregulate RRM2 mRNA levels in xenograft AML mice, acting as a specific and potent RRM2 inhibitor.^61^ In combination, AZA with pevonedistat showed significant synergy in the treatment of AML cell lines and AML xenografts compared to a single agent and decreased levels of RRM2 expression.^62^

## Safety and clinical activity of pevonedistat + AZA

An open-label Phase 1b clinical trial (NCT01814826) investigating combination pevonedistat plus AZA in treatment-naïve AML was performed with the primary objective focused on safety and tolerability and secondary objectives noting PK and disease response. Patients included were ⩾60 years of age with newly diagnosed untreated AML and unfit for induction therapy. Pevonedistat was given in escalating doses starting at 20 mg/m^2^ IV on days 1, 3, and 5 with AZA 75 mg/m^2^ either IV or SQ on days 1 to 5, 8, 9 every 28 days. The study included both *de novo* AML (36/64, 56%) and secondary AML (28/64, 44%). Owing to two of three patients experiencing a DLT at 30 mg/m^2^, the MTD for pevonedistat was determined to be 20 mg/m^2^ with standard AZA dosing (75 mg/m^2^). The most common AEs were constipation (48%), fatigue (42%), nausea (42%), and anemia (39%). Febrile neutropenia (30%) and anemia (30%) were the most common grade ⩾3 AEs. Overall, combination therapy was well tolerated, with 6.2% (4/64) of patients stopping therapy due to transaminitis and febrile neutropenia with no death attributed to pevonedistat. Pevonedistat PK was similar and was not affected by concurrent AZA when compared to historical single-agent data. The ORR was 50% (CR: 31%; CRi: 8%; PR:11%). Many patients achieved their responses in 2–4 cycles of treatment (63% and 91%, respectively). Median OS of the MTD cohort was 7.0 months with 6-month and 12-month survival 52% and 45%, respectively. The ORR and median OS was 46% (CR 29%, CRi 7%, and PR 11%) and 5.6 months in secondary AML, respectively, compared to 53% ORR (CR 33%, CRi 8%, and PR 11%) and 11.2 months median OS in *de novo* AML. Notably, those who did achieve CR had improved OS of 18.8 months compared to 8.3 months for those with CRi/PR. Although this phase 1b study was not designed or powered to compare clinical activity with historical control groups, these overall findings were encouraging in this older AML patient population who were unfit for intensive chemotherapy. For example, in AZA-AML-001, a randomized phase 3 study of AZA *versus* physician choice of best supportive care, low-dose cytarabine or induction chemotherapy in ⩾65-year-olds with newly diagnosed AML (BM blasts > 30%), ORR was 27.8% (CR = 19.5%; CRi = 8.3%), and median OS was 10.4 months in the AZA arm.^63^

Promising results from the phase 1b combination of AZA and pevonedistat in untreated AML patients led to the multicenter phase 2, randomized, controlled, open-label trial (NCT02610777) comparing AZA and pevonedistat *versus* single-agent AZA for higher-risk MDS/CMML and low-blast AML (BM blasts 20–30%) previously not treated with an HMA. Patients were randomized 1:1 with pevonedistat 20 mg/m^2^ on days 1, 3, and 5 and AZA 75 mg/m^2^ on days 1–5, 8, and 9 *versus* single-agent AZA at the same dose and schedule. Patients were stratified into low-blast AML and MDS/CMML with IPSS-R risk of intermediate, high, and very high risk. The study was initially designed with a primary endpoint of event-free survival (EFS) though this was subsequently amended after regulatory feedback to include OS as primary endpoint and EFS as a secondary endpoint. Overall, 120 patients were enrolled, with 58 patients in the AZA with pevonedistat arm and 62 patients in the AZA arm. The median OS was 21.8 months *versus* 19 months with AZA plus pevonedistat *versus* AZA, respectively, which did not reach statistical significance (*p* = 0.33). The median EFS showed a trend toward improvement with AZA plus pevonedistat (21.0 months *versus* 16.6 months, respectively) though also did not reach statistical significance (*p* = 0.076). In addition, for those who could be evaluated for response, ORR was 70.9% *versus* 60.4% for AZA with pevonedistat and AZA, respectively, with 7 months longer median duration of response for combination therapy. On subset analysis, higher-risk MDS patients had a non-significant improvement in median OS of 23.9 months with combination therapy compared with 14.8 months for AZA alone (*p* = 0.24) with significantly improved EFS of 20.2 months *versus* 14.8 months, respectively [hazard ratio (HR): 0.539, *p* = 0.045]. Higher-risk MDS also had improved ORR’s with combination therapy (79.3% *versus* 56.7%; CR rate of 51.7% *versus* 26.7%, and duration of response 34.6 *versus* 13.1 months). In terms of safety, there was no significant difference between reported AEs between pevonedistat and AZA *versus* AZA alone, with the most common grade ⩾3 AEs being neutropenia (33% *vs* 27%), febrile neutropenia (26% *vs* 29%), anemia (19% *vs* 27%), and thrombocytopenia (19% *vs* 23%). In addition, patient health-related quality of life surveys were similar between the two treatment groups suggesting that the addition of pevonedistat did not lead to worsening quality of life and symptom burden.^64,65^

Although not a primary or secondary endpoint, this study investigated clonal emergence through DNA sequencing of BM aspirations collected during the study. In total, 96 BM aspirations were collected at baseline, and 58 longitudinal marrows were sampled during the treatment of 33 high-risk MDS, 7 CMML, and 18 low-blast AML patients. Pevonedistat with AZA showed significantly fewer treatment-emergent mutations, 29.3% *versus* 49.6%, compared to AZA alone. This suggests that combination therapy reduces the mutational burden and possibly decreases the chance to develop treatment-resistant mutations or disease progressing mutations, though more study is warranted in this setting.^66^

Although the randomized phase 2 study did not meet the primary endpoint of the study, the encouraging results overall led to the design of the PANTHER study (NCT03268954), a multicenter, randomized, open-label phase 3 study investigating the combination of pevonedistat plus AZA *versus* single-agent AZA in first-line treatment of higher-risk MDS, CMML and low-blast AML. The primary endpoint of the PANTHER study was EFS, defined as time to death or transformation to AML in higher-risk MDS/CMML and time to death in low-blast AML. This international study enrolled 472 patients randomized in a 1:1 fashion with pevonedistat 20 mg/m^2^ on days 1, 3, and 5 and AZA 75 mg/m^2^ on days 1–5, 8, and 9 (*n* = 227) *versus* single-agent AZA (*n* = 227) at the same dose and schedule. Results of this study were presented at the annual American Society of Hematology (ASH) conference in December 2021 in Atlanta, GA.^67^ Median age was 73 and 74 years in the combination arm *versus* AZA, respectively. Among the higher-risk MDS cohort (pevonedistat plus AZA: *n* = 161; AZA: *n* = 163), there was a similar proportion of patients with intermediate, high, and very high risk among both arms. In addition, there was a balanced distribution of prognostic gene mutations among both arms with the most frequent mutations being *ASXL1* (40.7% *versus* 39.3%), *TET2* (30.4% *versus* 25.9%), *TP53* (28.9% *versus* 25.9%), *RUNX1* (25.9% *versus* 32.6%), *SRSF2* (27.4% *versus* 20.7%), *DNMT3A* (20.7% *versus* 15.6%), and *STAG2* (18.5% *versus* 20.7%) in combination arm *versus* AZA, respectively. In the intent-to-treat (ITT) population, the median EFS (17.7 months *versus* 15.7 months; *p* = 0.56) and OS (20.3 months *versus* 16.8 months; *p* = 0.18) were not significantly different between both arms. In the higher-risk MDS cohort, median EFS was 19.2 months *versus* 15.6 months (*p* = 0.43) in the combination arm *versus* AZA alone, respectively, whereas median OS was non-significantly longer with the combination arm in higher-risk MDS (21.6 months *versus* 17.5 months, respectively; *p* = 0.092). In addition, ORR was similar in both arms in the ITT population (combination arm: 28% *versus* AZA: 32%) without significant differences in any disease subgroups. Prespecified subgroup analysis only identified a significant improvement in OS in males with combination therapy, whereas all other subgroups showed no significant differences in OS between both arms. Finally, there was a significant improvement in OS in the combination arm in patients who received more than six cycles of therapy overall (median OS = 27.1 months *versus* 22.5 months, respectively; *p* = 0.008). These sobering results were disappointing as the combination of pevonedistat plus AZA did not significantly improve overall clinical outcomes compared with AZA alone. Given the rigorous study design to maintain dose intensity and mitigate dose reductions of AZA, it is possible that any additive impact of pevonedistat was diminished in this patient population. Nonetheless, further study is warranted to identify biomarkers and/or subgroups of patients who may benefit from the combination of pevonedistat plus AZA in MDS, CMML, and AML.

## Future directions with pevonedistat

Although pevonedistat combined with AZA did not reach its primary outcome for EFS, there is still a strong rationale for novel combination strategies with pevonedistat and other therapeutic agents in MDS and AML. This section will explore other encouraging therapy combinations and future directions yet to be explored in the use of pevonedistat (Table 1).

**Table 1.** Ongoing combination studies of pevonedistat.

Study identifier	Study name	Drug name	Combination therapy	Clinical phase	Indication	Primary endpoints	Enrollment	Sponsor	Study start	(Estimated) Completion Date	Status
NCT03009240	Pevonedistat and Decitabine in Treating Patients With High Risk Acute Myeloid Leukemia	Pevonedistat	Decitabine	I	Newly diagnosed AML not eligible for intensive chemotherapy or relapsed/refractory AML	Phase I: MTD, DLT, adverse effect	30	City of Hope Medical Center	2017	2021	Active, not recruiting
NCT03459859	Pevonedistat and Low Dose Cytarabine in Adult Patients With AML and MDS	Pevonedistat	Cytarabine	I	Relapsed/refractory AML or relapsed/refractory MDS	Phase I: MTD, safety	12	Justin Watts, MD, University of Miami	2018	2021	Completed
NCT03745352	Pevonedistat With Azacitidine Versus Azacitidine Alone in Treating Patients With Relapsed or Refractory Acute Myeloid Leukemia	Pevonedistat	Azacitidine	II	Relapsed/refractory AML	Phase II: OS	72	National Cancer Institute	2019	2022	Active, not recruiting
NCT03772925	Pevonedistat and Belinostat in Treating Patients With Relapsed or Refractory Acute Myeloid Leukemia or Myelodysplastic Syndrome	Pevonedistat	Belinostat	I	Relapsed/refractory AML or relapsed/refractory MDS	Phase I: MTD	30	National Cancer Institute	2019	2022	Recruiting
NCT03813147	Pevonedistat, Azacitidine, Fludarabine Phosphate, and Cytarabine in Treating Patients With Relapsed or Refractory Acute Myeloid Leukemia or Myelodysplastic Syndrome	Pevonedistat	Azacitidine, Fludarabine Phosphate, and Cytarabine	I	Relapsed/refractory AML or relapsed/refractory MDS	Phase I: MTD, DLT, pharmacokinetics, pharmacodynamics	12	National Cancer Institute	2019	2022	Active, not recruiting
NCT03862157	Azacitidine, Venetoclax, and Pevonedistat in Treating Patients With Newly Diagnosed Acute Myeloid Leukemia	Pevonedistat	Azacitidine, Venetoclax	I/II	Newly diagnosed s-AML or newly diagnosed CMML/MDS or post-HMA failure CMML/MDS	Phase I: DLT Phase II: CR/ CRi/ PR/ HI/ mCR	40	M.D. Anderson Cancer Center	2019	2024	Recruiting
NCT04090736	Study to Compare Azacitidine Plus Pevonedistat Versus Azacitidine in Patients With Acute Myeloid Leukemia Not Eligible for Standard Chemotherapy (PEVOLAM)	Pevonedistat	Azacitidine	III	Newly diagnosed AML not eligible for intensive chemotherapy	Phase III: OS	466	PETHEMA Foundation	2019	2023	Recruiting
NCT04172844	Pevonedistat, Azacitidine (or Decitabine), and Venetoclax for the Treatment of Patients With Acute Myelogenous Leukemia (PAVE)	Pevonedistat	Azacitidine, Venetoclax	I	Relapsed/refractory AML (dose-escalation phase), newly diagnosed AML or relapsed/refractory AML (expansion phase)	Phase I: MTD, adverse events	24	Medical College of Wisconsin	2020	2024	Active, not recruiting
NCT04266795	Triple Combination of Pevonedistat and Venetoclax Plus Azacitidine in Adults With Acute Myeloid Leukemia Who Are Unfit for Intensive Chemotherapy (PEVENAZA)	Pevonedistat	Azacitidine, Venetoclax	II	Newly diagnosed AML not eligible for intensive chemotherapy	Phase II: EFS	164	Takeda	2020	2024	Active, not recruiting
NCT04712942	Treatment of MDS/AML Patients With an Impending Hematological Relapse With AZA or AZA and Pevonedistat	Pevonedistat	Azacitidine	II	AML or MDS after first CR with intensive chemotherapy or CR after alloSCT with MRD	Phase II: MRD status after 3 months of treatment	102	University of Leipzig	2021	2025	Recruiting

AML, acute myeloid leukemia; CMML, chronic myelomonocytic leukemia; CR, complete remissions; DLT, dose-limiting toxicity; EFS, event-free survival; HI, hematologic improvement, HMAs, hypomethylating agents; mCR, marrow complete response; MDS, myelodysplastic syndrome; MRD, measurable residual disease; MTD, maximally tolerated dose; OS, overall survival; PR, partial remissions.

In normal cells, the apoptotic pathway is regulated by extrinsic and intrinsic pathways with tight control of the intrinsic pathway regulated by the B-cell lymphoma-2 (BCL2) protein family.^68,69^ FDA-approved venetoclax, a highly selective BCL2 inhibitor, changed the treatment paradigm forming a new standard-of-care for newly diagnosed older adults with AML who are ineligible for intensive chemotherapy. Those treated with combination venetoclax and AZA had a median OS of 14.7 months and CR/CRi of 66.4% compared to a median OS of 9.6 months and CR/CRi of 28.3% with AZA alone leading to a new standard-of-care in this patient population.^70^ Despite the significant improvement responses and survival with AZA and venetoclax, resistance to venetoclax is common and thought to be mediated by anti-apoptotic proteins like myeloid cell lymphoma-1 (MCL1) and B-cell lymphoma-extra large (BCL-xL), which are part of the BCL2 family.^71^ The BCL2 family also includes proapoptotic proteins that can be divided into two subtypes based on their structure: multidomain proteins (e.g. BAX and BAK) and BH3-only proteins (e.g. NOXA, PUMA, BIK, BIM).^72^ The combination of venetoclax and AZA arose from studies that demonstrated HMAs downregulate MCL1 expression working synergically with venetoclax to inhibit BCL2 to kill AML cells.^73^ It has been demonstrated that the expression ratio of BCL2 family anti- to proapoptotic proteins may dictate sensitivity to venetoclax though determining optimal biomarkers for response (and resistance) to AZA and venetoclax in AML remains an area of active investigation.^74^

Prior preclinical studies have shown that pevonedistat and AZA both upregulate NOXA, which competes with effector molecules at the BH3 binding-site of MCL1 and inhibits its anti-apoptotic function allowing for activation of BAX/BAK and subsequent apoptosis.^75–77^ It was recently demonstrated that pevonedistat combined with AZA synergistically induces NOXA expression more than either single agent alone in AML cells. This finding led to the exploration of triple combination therapy pevonedistat, AZA, and venetoclax in an AML xenograft murine model. Strikingly, triplet therapy had the greatest tumor growth inhibition compared to doublet or singlet treatment groups.^78^ These findings, coupled with the acceptable safety profile of pevonedistat and its promising preclinical synergy with AZA plus venetoclax, led to the investigation of a triplet (AZA plus venetoclax and pevonedistat) therapeutic approach in newly diagnosed AML.

A phase I/II, open label, clinical trial initiated by M.D. Anderson Cancer Center investigated triplet therapy safety and clinical activity in newly diagnosed secondary AML patients unfit for intensive chemotherapy. The trial is estimated to enroll 40 patients and the preliminary data of this study was presented at the European Hematology Association (EHA) on 12 June 2020.^79^ At the time of the poster release, 10 patients had been enrolled to the phase I arm and two patients to the phase II arm. The phase I arm investigated DLTs associated with AZA 75 mg/m^2^ on days 1–7, venetoclax on days 1–28 for cycle 1 and days 1–21 for cycle 2 and beyond (doses ranged from 200 to 400 mg daily), and pevonedistat 20 mg/m^2^ IV on days 1, 3, and 5 every 28-days. Myelosuppression was similar to what was seen historically with venetoclax and AZA. Common grade ⩾3 adverse events included hypophosphatemia (60%), infection (50%), febrile neutropenia (20%), and nausea/vomiting (20%). There were two deaths unrelated to treatment. The responses for patients included CR in 50% (5/10), CRi in 10% (1/10), MLFS in 10% (1/10), no response in 20% (2/10), and one early death. These results were especially promising given that 3 of 4 patients with *TP53* and 3 of 5 patients with complex cytogenetics achieved a CR/CRi. An update of this study was presented at the 2021 ASH Conference.^80^ Twenty-eight patients were treated with AZA, venetoclax, and pevonedistat with 3/28 patients receiving venetoclax 200 mg daily and 25/28 patients receiving venetoclax 400 mg daily. Non-hematologic grade 3 adverse events were reported to be infection/neutropenic fever in 61% (18/28), hypophosphatemia in 29% (8/28), hyperglycemia/hyperbilirubinemia/increased AST or ALT in 11% (3/28), pneumonitis/acute kidney injury/hypokalemia/vomiting in 7% (2/28) of patients. The ORR and CR + CRi rate was 71% (20/28) and 64% (18/28), respectively. For the patients who achieved CR, 44% (8/18) obtained MRD negativity by flow cytometry. The median OS was 8.2 months and median RFS was 7.5 months in this cohort of patients.

Based on the phase I arm data, the recommended phase 2 dosing of this triplet regimen was determined to be AZA 75 mg/m^2^ on days 1–7, pevonedistat 20 mg/m^2^ IV on days 1, 3, and 5 every 28-days, and venetoclax 400 mg on days 1–28 with initial ramp-up for cycle 1 and days 1–21 if confirmed CR was reached for cycle 2 and beyond. This regimen established the dosing schedule for the Takeda-sponsored randomized, open-label, controlled, phase 2 study of pevonedistat, venetoclax, and azacitidine *versus* venetoclax plus azacitidine (PEVENAZA) in newly diagnosed AML who were unfit for intensive chemotherapy, which is being conducted globally (NCT04266795). Unfortunately, this study was recently terminated early and closed to accrual. Results of this study are eagerly awaited. The Medical College of Wisconsin is also conducting a phase 1b dose-escalation clinical trial (NCT04172844) to evaluate the safety of pevonedistat when given with AZA and venetoclax with results yet to be presented or published.

## Conclusion

As it currently stands, HMA monotherapy remains the standard of care and recommended first-line treatment for MDS. The addition of pevonedistat to AZA did not improve clinical outcomes of EFS and OS compared with AZA alone in high-risk MDS.^20,81^ However, it must be highlighted that the clinical data behind pevonedistat is still in its infancy. Further investigation into the mechanisms behind pevonedistat effects on cancer cells will lead to new combination therapies for future clinical trials. Pevonedistat is a promising agent to add in combination due to its acceptable safety profile and effects on multiple cellular regulatory pathways critical to cancer survival. HMA’s will likely remain the backbone of MDS treatment for the foreseeable future. However, novel combination strategies with putative biomarkers for patients with distinct genomic profiles are the future of MDS therapy, whereby a precision medicine-based approach will hopefully lead to a paradigm shift in the management of these patients.

## Declarations

**Ethics approval and consent to participate:** Not applicable.

**Consent for publication:** Not applicable.

**Availability of data and materials:** Not applicable.
